# Patterns of admission and outcome of patients admitted to the intensive care unit of Addis Ababa Burn Emergency and Trauma Hospital

**DOI:** 10.1038/s41598-023-33437-z

**Published:** 2023-04-19

**Authors:** Dirijit Mamo, Etsegenet Aklog, Yemane Gebremedhin

**Affiliations:** Department of Emergency and Critical Care Medicine, St. Paul`S Hospital Millennium Medical College, Addis-Ababa Burn, Emergency, and Trauma Hospital, Addis-Ababa, Ethiopia

**Keywords:** Health care, Medical research

## Abstract

Data on patterns of intensive care unit (ICU) admission including age, and severity of illness is essential in developing better strategies for resource allocation to improve outcomes. A 2-year cross-sectional study of 268 patients using a systematic random sampling and structured questionnaire obtained from the database was conducted with the aim of examining patterns of admission among patients admitted to the ICU of Addis Ababa burn emergency and trauma (AaBET) hospital. Data were entered into Epi-Info version 3.5.3 and exported to SPSS version 24 for analysis. Bivariate and multivariate logistic regression were used for association. A P-value of 0.05 at a 95% confidence interval was declared clinically significant. Of the 268 charts reviewed, 193 (73.5%) of them were men with a mean age of 32.6 years. Trauma accounted for 163 (53.4%) of admissions. Burn admission category, Glasgow coma score of 3–8, and not receiving pre-referral treatment were found to be substantially correlated with mortality in both bivariate and multivariate analysis. Trauma constituted a sizeable cause of ICU admission. Road traffic accidents of traumatic brain injuries were the major causes of admission. Developing good pre-referral care equipped with manpower and ambulance services will improve the outcome.

## Introduction

An intensive care unit is a specialized inpatient unit that provides continuous monitoring and cares for those patients who are critically ill and need sophisticated materials and multidisciplinary human resources. These units are extremely expensive in terms of manpower and equipment utilization. In developed nations like the United States, this unit consumes 15–40% of the entire hospital cost^1,2^.

The management of individuals who are critically ill places a great deal of emphasis on intensive care. Critical patients who need sophisticated airway, respiratory, and hemodynamic support are typically transferred to the intensive care unit (ICU), where they receive advanced treatment, and both invasive and noninvasive monitoring to achieve a better outcome^1,3^.

Access to critical care is a crucial component of the health care system; hence, critically ill patients are admitted to the ICU to reduce mortality and morbidity. Mortality in the ICU is a huge burden on the economy of the country. According to the world health organization (WHO) manual of surgical care referral, hospitals should have intensive care units capable of surgical intervention^4^.

Timely and appropriate care is the key to achieving good outcomes in acutely ill patients, but the effectiveness of critical care may be limited in resource-limited settings. Specialty-trained staff and standardized processes of care such as checklists are frequently lacking in ICU setups in low and middle-income countries, which in turn results in poor patient outcomes. As in the study done in Tanzania with increased mortality among patients with traumatic brain injury^5^.

The burden of critical illness in low-income countries is high and expected to rise. In Ethiopia, there are approximately 0.3 public ICU beds per 100,000 people. Services were concentrated in the capital, Addis Ababa, with 25% of bed capacity and 51% of critical care physicians. No ICU had piping for oxygen. Only 106 (33%) of the beds had all three of the basic recommended noninvasive monitoring devices (sphygmomanometer, pulse oximetry, and electrocardiography). There was limited capacity for ventilation (n = 189; 58%), invasive monitoring (n = 9; 3%), and renal dialysis (n = 4; 8%). Infection prevention and control strategies were lacking^6^.

In Ethiopia, basic information on the pattern of admission and outcome of patients admitted to multidisciplinary ICUs is lacking to our best knowledge. Hence, the aim of this study is to solicit, information on the patterns of admission, outcome, and associated factors (factors that influence the outcome of patients in ICU including age, the severity of their illness, prehospital care prior to patient transfer comorbidities, etc.) of patients admitted to Addis Ababa burn, emergency and trauma hospital.

## Methods and materials

### Study area and period

This research was carried out at the Addis Ababa Burn, Emergency, and Trauma hospital (AaBET), which is situated in Addis-Ababa, Ethiopia, the nation's capital. It was founded on July 15, 2015, as a part of St. Paul Hospital Millennium Medical College (SPHMMC), the second-largest hospital in the nation, AaBET hospital is unique in its ability to care for any urgent patient while placing a specific focus on burn and trauma patients.

### Study design

An institutional-based cross-sectional study over a period of two years was conducted by reviewing the charts of admissions made to AaBET ICU from September 2017 to August 2019.

### Source population

Records of all patients admitted to the AaBET ICU from September 2017 to August 2019.

### Study population

All sampled patient records admitted to the AaBET ICU over a period of two years from September 2017 to August 2019 were included.

### Eligibility criteria

#### Inclusion criteria

Patients admitted to AaBET ICU during the study period were eligible and included in this study, including pediatric age groups admitted due to trauma, burn, and non-traumatic neurosurgical conditions.

#### Exclusion criteria

Pediatric patients with a medical condition, neonates, and those patients with incomplete records were excluded. The pediatric and neonatal age groups are excluded from this study as there is a different ICU setup in SPHMMC providing service for this specific group.

### Sample size determination and sampling procedure

The final sample size was determined to include 268 patients. A single population proportion formula and systematic random sampling method were used.

### Data collection tool and procedure

A structured questionnaire that contains socio-demographic factors, patterns, sources of admission, and patient outcomes was prepared. Necessary data was extracted by reviewing the patient’s chart.

### Data quality control

The data collection tool was pretested on 5% of samples on similar study subjects admitted in August 2016 for the validity of the survey tool and questionnaires were standardized. Data collectors were trained for one-day and daily supervision of data was done by the principal investigator and completeness was checked throughout.

### Data processing and analysis

The data were cleared, coded, and entered into epi info Version 3.5.3 and exported to SPSS version 24 for analysis. Descriptive statistics including frequency, percentage, and mean were derived. Crude odds ratio (COR) and adjusted odds ratio (AOR) was analyzed with a 95% confidence interval (CI). Binary logistic regression was used to see the association between dependent and independent variables. Those variables with a P-value of ≤ 0.05 on binary logistic regression were further considered for multivariate logistic regression to determine the independent associations of each variable and control confounding variable. P-value < 0.05 was declared a statistically significant association.

### Study variables

#### Dependent variables

Outcome (alive, and expired).

#### Independent variables

Age, sex, length of stay, diagnosis at admission, disease characteristics (medical vs surgical, infectious vs non–infectious), source of admission, and pre-referral treatment.

### Ethical consideration

Ethical clearance and approval to conduct this research were obtained from St. Paul’s hospital millennium medical college institutional review board (IRB). To ensure confidentiality names and other personal identities were not used during data collection and manuscript writing. The need for informed consent for research and publication was waived by SPHMMC IRB due to the retrospective nature of the study with Ref. No. of pm 21/384.

The authors declare that all the methods included in the study are in accordance with the declaration of Helsinki**.**

## Results

### Sociodemographic characteristics of patients admitted to the ICU

From a total of 268 studied patient charts 193 (73.5%) were male and 75 (26.5%) were females. The Patients’ age ranged from 2 to 78. The mean age of the patients was 32.6 years. The majority 79 (29.5%) belong to the age group 20–29 years. About half of the patients 128 (47.8%) were from the Oromia region.

The majority 188 (70.1%) use the ambulance as a means of transportation upon arrival to AaBET emergency department prior to their admission to the ICU and 177 (66%) patients were triaged to the red area (Table 1).

**Table 1:** Mode of transportation and triage category of patients up on arrival to ED before admission in ICU of AaBET Hospital, Addis Ababa Ethiopia, Sept 2017–Aug 2019 (*n = 268).

	Male		Female		Total (%)
	No	%	No	%	
Mode of transportation					
Taxi	31	11.6	7	2.6	14.2
Ambulance	132	49.3	56	20.9	70.1
Private Car	17	6.3	2	0.7	7.1
Other	17	6.3	6	2.2	8.6
Triage category					
Red	126	47.0	51	19	66
Orange	48	17.9	13	4.9	22.8
Yellow Green	23	2.6	7	2.6	11.2

*ED* Emergency Department.

### Patterns of admission categories

The most common reasons for admission to the ICU were Trauma 163 (53.4%), followed by Medical 66 (24.6%), Non-traumatic neurosurgical conditions 22 (8.2%), and Burn 17 (6.3%).

**Figure 1:**
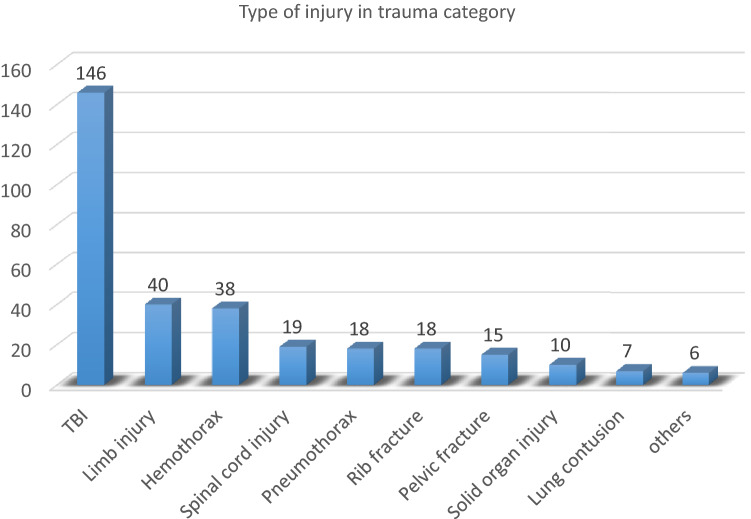
Distribution of Trauma categories admitted in ICU of AaBET Hospital, Addis Ababa Ethiopia, Sept 2017–Aug 2019 (*n = 163). *Number of patients, *TBI* Traumatic Brain Injury.

In the Trauma category, the major subcategory was traumatic brain injury (TBI) 146 (82.5%) of the cases, followed by limb injury, in 40 (14.9%) (Fig. 1).

The major 104 (59.1%), cause of injury was road traffic accidents followed by falling from height 33 (18.8%). Of the total 146 TBI patients 104 (71.2%) were sever TBI, 29 (20%) moderate TBI, and 13 (8%) mild TBI. The computed tomography finding of TBI patients showed, 46 (31.9%) had a diffuse axonal injury, and 41 (28.5%) had Epidural Hematoma. Moreover, 70% of TBI patients underwent a surgical procedure, out of which 49 (70%) was hematoma evacuation, 17 (24.3%) depressed skull fracture elevation, 14 (20%) decompressive craniectomy, and 4 (5.7%) received other neurosurgical procedures. Of overall TBI patients 73 (77.7%) were intubated upon arrival to the ICU for air-way protection (Table 2).

**Table 2:** Distribution of trauma patients based on mechanism of injury among patients admitted in AaBET hospital ICU, Addis Ababa Ethiopia, Sept 2017–Aug 2019 (*n = 163).

	RTA No. (%)	Falling down No. (%)	Gun shoot No. (%)	Assault No. (%)	Others No. (%)
Sex					
Male	93 (57.1)	28 (17.1)	0	19 (11.65)	12 (7.4)
Female	7 (4.3)	4 (2.45)	0	0	0
Affected organ system					
TBI	90 (55.2)	26 (15.95)	8 (4.9)	20 (12.27)	2 (1.2)
Solid injury	7 (4.29)	0	0	0	0
Rib fracture	14 (8.59)	4 (2.45)	0	0	0
Hemothorax	31 (19.02)	2 (1.2)	0	0	2 (1.2)
Pneumothorax	15 (9.2)	3 (1.8)	0	0	0
Lung contusion	6 (3.68)	0	0	0	0
Limb injury	29 (17.8)	11 (6.74)	0	0	0
Pelvic fracture	12 (7.36)	0	0	0	0
Spinal injury	14 (8.59)	3 (1.8)	0	0	0
Other	5 (3.06)	2 (1.2)	0	0	0

There were a total of 17 burn patients admitted to the ICU during the study period. The most common etiology for all burn admissions was electrical burn. The reason for admission to the ICU was respiratory failure 10 (58.8%) and 7 (41.1%) were admitted due to Septic shock.

Non-traumatic neurosurgical conditions accounted for 22 (8.2%). Out of which, 10 (45.5%) were admitted due to Astrocytoma. 15 (68%) patients underwent tumor debulking and extirpation, 5 (22.7%) external ventricle drain (EVD) insertions, and 3 (13.6%) patients underwent ventricle-peritoneal (VP) shunt insertion for an indication of obstructive hydrocephalus was done. The main reason for admission after the procedures were Respiratory failure 50%, followed by Patient intubation due to a low glass-gow coma score (GCS) which was 7 (31.8%).

Of the total 66 medical cases, the majority were admitted for neurologic conditions 23 (34.8%), which include stroke, infectious intracranial space-occupying lesion (ICSOL), and encephalopathies. Infectious causes were the second most common causes of medical admission to ICU which accounted for 19 (28.78%), followed by non-infectious causes of respiratory failure 11 (16.6%), and cardiovascular and endocrine conditions 7 (10.6%) and 6 (9.09%) respectively.

### ICU treatment

The majority of patients in ICU 245 (94.2%) were treated with antibiotics, 243 (93.5%) received mechanical ventilation support, and 146 (56.2%) received hemodynamics support.

### ICU related complications

**Figure 2:**
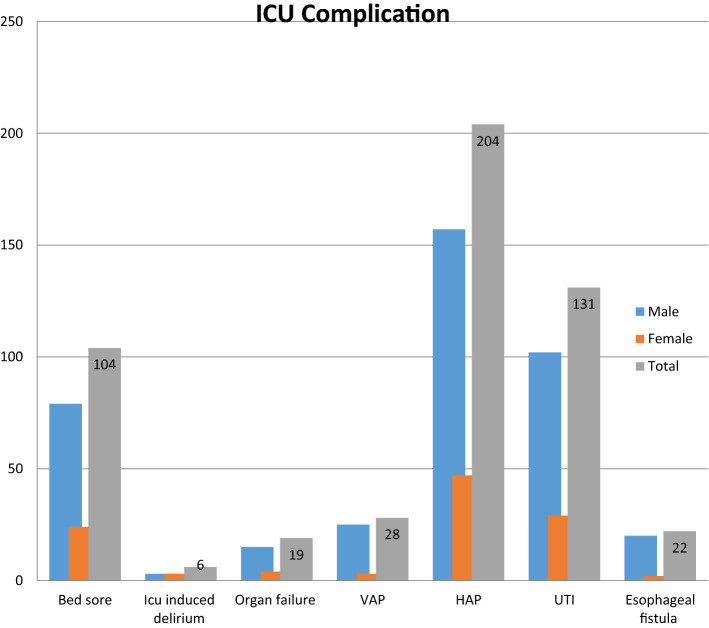
Distributions of ICU associated complications among patients admitted to AaBET Hospital ICU, Addis Ababa Ethiopia, Sept 2017–Aug 2019 (*n = 268). *HAP* Hospital Acquired Pneumonia, *UTI* Urinary Tract Infection, *VAP* Ventilator Associated Pneumonia.

The majority of patients developed HAP 204 (77.1%) followed by UTI 131 (48.8%) and Bed sore 104 (39.5%) (Fig. 2, Table 3).

**Table 3:** Distribution of patients complication on the bases of ICU length of stay, and days on Mechanical Ventilator in ICU of AaBET Hospital, Addis Ababa Ethiopia, Sept 2017–Aug 2019 (*n = 268).

	Bedsore	Delirium	Organ failure	VAP	HAP	UTI	Esophageal fistula
Length of ICU stay							
< 1	0	0	0	0	0	0	0
1–5	0	0	2 (0.8%)	2 (0.8%)	4 (1.5%)	2 (0.8%)	0
11–15	0	0	2 (0.8%)	0	35 (13.3%)	22 (8.4%)	0
16–20	4 (1.5%)	0	0	6 (2.3%)	30 (11.4%)	12 (4.6%)	2 (0.8%)
21–25	10 (3.8%)	0	0	0	15 (5.7%)	10 (3.8%)	0
26–30	10 (3.8%)	3 (1.1%)	0	3 (1.1%)	19 (7.2%)	11 (4.2%)	2 (0.8%)
> 30	80 (30.4%)	3 (1.1%)	15 (5.7%)	17 (6.5%)	80 (30.4%)	63 (24%)	18 (6.8%)
Days on a mechanical ventilator							
1–15	7 (2.8%)	0	4 (1.5%)	8 (3.2%)	83 (32.8%)	41 (16.2%)	4 (1.5%)
16–30	56 (22.1%)	3 (1.2%)	4 (1.5%)	8 (3.2%)	73 (28.9%)	39 (15.4%)	3 (1.1%)
31–45	19 (7.5%)	0	7 (2.8%)	8 (3.2%)	17 (2.8%)	19 (7.5%)	3 (1.1%)
46–60	13 ( (5.1)	3	0	0	15 (5.9%)	15 (5.9)	8 (3.2%)
61–75	7 (2.8%)	0	4 (1.5%)	4 (1.5%)	7 (2.8%)	7 (2.8)	4 (1.5%)
75–90	2 (0.8%)	0	0	0	2 (0.8%)	2 (0.8%)	0

### The outcome of ICU-admitted patients

During the study period, 268 patients were admitted to ICU, from these patients 94 (35.1%) patients were transferred to their respective wards, and 91 (34.1%) were discharged home. The overall ICU mortality was 58 (21.6%). Among patients of all age groups, shock, brain herniation, and septicemia were conditions that resulted in the highest in-ICU mortality rate of over 80%. Severe TBI and tetanus had the longest median ICU stay, with in-ICU mortality of 71.1% (Table 4).

**Table 4:** Outcome of patients based on the mode of transportation, place of admission, and length of ICU stay at ICU of AaBET Hospital, Addis Ababa Ethiopia, Sept 2017–Aug 2019 (*n = 268).

	Died No. (%)	Left AMA No. (%)	Ward admission No. (%)	Referred No. (%)	Discharged No. (%)
Mode of transportation					
Taxi	13 (4.9)	0	14 (5.2)	2 (0.7)	9 (3.4)
Ambulance	38 (14.2)	0	58 (21.6)	23 (8.6)	69 (25.7)
Private car	2 (0.7)	0	10 (3.7)	0	7 (2.6)
Other	5 (1.9)	0	9 (3.4)	0	9 (3.4)
Place of admission					
ED	29 (10.8)	0	43 (16)	12 (4.5)	56 (20.9)
Neurosurgery ward	0	0	11 (4.1)	0	0
Orthopedics ward	0	0	2 (0.7)	0	5 (1.9)
PACU	12 (4.5)	0	33 (12.3)	13 (4.9)	30 (11.2)
Burn unit	13 (4.9)	0	2 (0.7)	0	3 (1.1)
Transferred to other facility	4 (1.5)	0	0	0	0
Days in ICU					
1–5 days	16 (6)	0	2 (0.7)	0	2 (0.7)
6–10	17 (6.3)	0	11 (4.1)	2 (0.7)	18 (6.7)
11–15	4 (1.5)	0	19 (7.1)	0	14 (5.2)
16–20	5 (1.9)	0	18 (6.7)	0	13 (4.9)
21–25	0	0	7 (2.6)	6	5 (1.9)
26–30	2 (0.7)	0	7 (2.6)	0	13 (4.9)
> 30	14 (5.2)	0	27 (10.1)	17 (6.3)	29 (10.8)
Admission category					
Trauma	28 (10.4)	0	61 (22.8)	17 (6.3)	57 (21.3)
Burn	8 (3)	0	0	0	9 (3.4)
Medical	21 (7.8)	0	20 (7.5)	4 (1.5)	21 (7.8)
Non-traumatic NS	1 (0.4)	0	10 (3.7)	4 (1.5)	7 (2.6)

*LMA* left against medical advice, *NS* Neurosurgical, *PACU* Post Anesthesia Care Unit.

### Determinants of ICU outcome

In order to investigate the association of independent variables with the outcome both bivariate; and multivariate analyses were used. In bivariate analyses; GCS of 3–8, admission category, pre-referral care which implies the act of stabilization and transportation of critically ill patients, and age were significantly associated with death.

In the multivariate analysis; GCS, admission category, and pre-referral care by referring institutions were retained as determinants of outcome. GCS 3–8 had 7.91 times [AOR = 7.91] 95% CI (2.9, 14.2) more likely to have died than a patient with GCS 14–15. Burn patients had 2.63 times [AOR = 2.63], 95% CI (1.39, 4.98)] more likely to die than other trauma (general surgical and orthopedic) patients. A patient who does not have pre-hospital care had 12.1 times [AOR = 12.1], 95% CI (3.7, 21.3)] more death than those who received pre-hospital care (Table 5).

**Table 5:** Multivariate analysis of factors associated with the outcome of patients admitted in ICU of AaBET Hospital, Addis Ababa Ethiopia, Sept 2017–Aug 2019 (*n = 268).

Variables	Death		COR (95% CI)	AOR (95% CI)	P-value
	Yes (*n = 58)	No (*n = 210)			
GCS					
3–8	42 (15.8	97 (36.5)	**3.94** (**1.8,8.3)**^**++**^	**7.91** (**2.9,** **14.2)**^**++**^	**< 0.001**
9–13	6 (2.3)	20 (7.5)	2.73 (0.88,8.38)	**10.0** (**2.2,** **44.2)**^**++**^	**0.003**
14–15	10 (3.8)	91 (34.2)	1	1	
Admission category					
Trauma	23 (8.6)	140 (52.2)	1	1	
Burn	6 (2.2)	11 (4.1)	**3.32** (**1.11,** **9.85)**^**++**^	**2.63** (**1.39,** **4.98)**^**+**^	**0.002**
Medical	21 (7.8)	45 (16.8)	**2.84** (**1.439)**^**++**^	**2.33** (**1.32,** **4.12)**^**+**^	**0.015**
Non-traumatic neurosurgical	8 (3)	14 (5.2)	**3.47** (**1.31,** **9.21)**^**++**^	**2.05** (**1.19,** **3.52)**^**+**^	**0.002**
Pre-referral care					
No	52 (19.4	98 (36.6)	**9.91** (**4.0,** **24.0)**^**++**^	**12.1** (**3.7,** **21.3)**^**++**^	**< 0.001**
Yes	6 (2.2)	112 (41.8	1	1	
Age (years)					
0–9	2 (0.7)	11 (4.1)	1	1	
10–19	4 (1.5)	25 (9.3)	0.88 (0.14, 5.53)	0.67 (0,71, 7,14)	0.642
20–29	6 (2.2)	68 (25.4)	0.48 (0.87, 2.71)	0.32 (0.04, 2.32)	0.260
30–39	5 (1.9)	50 (18.7)	0.55 (0.09, 3.21)	0.45 (0.06, 3.36)	0.440
40–49	8 (3)	17 (6.3)	2,58 (0.46,14.52)	1.23 (0.16, 9.17)	0.840
40–59	9 (3.4)	21 (7.8)	2.35 (0.43, 12.8)	1.39 (0.2, 9.75)	0.737
60–69	10 (3.7	8 (3)	**6.8** (**1.17,** **20.3)**^**+**^	7.41 (0.88, 31.87)	0.064
70–80	14 (5.2)	10 (3.7)	**7.7** (**1.39,** **22.68)**^**+**^	4.47 (0.62, 21.90)	0.135

Significance values are in bold.

*Number of patients.

^**+**^Association on bivariate analysis.

^**++**^Significant Association on multivariate analysis.

*AOR* Adjusted Odds Ratio, *CI* Confidence Interval, *COR* Crude Odds Ratio.

## Discussion

This study assesses the pattern of disease, outcome, and associated factors among patients admitted to AaBET intensive care unit. Accordingly, trauma was the common cause of admission to the unit, and from all traumatic cases, TBI holds the upper hand in the admission category. Moreover, the majority of patients with initial low GCS died, and the overall ICU TBI mortality was 21.9% with mortality for severe TBI at 15.8%. This was similar to a study conducted at Jimma University specialized hospital (21.2%), in Ethiopia. Whereas, the mortality rate in severe head injuries was 57.9%, with initial GCS and the GOS (the Glass outcome scale) of all patients having a significant correlation with the outcome. However, the mortality rate for severe TBI in our study was lower than the study done in Jimma^7^. This could be due to the availability of diagnostic facilities and enough manpower for timely surgical intervention of such patients.

RTA was the major cause of injury for trauma patients in this study, compared with other studies conducted in Tanzania, Kenya, and other studies done in TASH, Ethiopia as well^8–10^.

The majority of patients admitted during the study period were young with the mean age at admission being 32.6 years, lower than similar studies conducted in developed nations^11–14^. But, compared with other studies conducted in Africa^13–16^. And, there was male predominance in our study, which was comparable to studies conducted in developing nations^8–10^. The overall young population in this study reflect the predominance of trauma as a cause of admission and most young male being the working force of the country had occupational exposure to trauma resulting in ICU admission. The other factor that might have contributed to the younger patient admission could be the result of prioritization of patients based on the outcome due to the limited existing resource in our center. However, decision-making on admitting patients was not addressed in this research. Assumptions were based on what’s being observed during our ICU and ED attachment period in AaBET hospital. However, further research is needed and a clear protocol needs to be developed to clarify issues regarding the admission criteria.

The overall in-ICU mortality rate in this study was 21.6%, which was lower than other studies in Tanzania which were 41.4%^8^. And, another study was conducted in TASH 31.5%^17^. And, less than a study conducted at Jimma University 32.2%^18^. This study attributed the high mortality to delayed presentation of patients as a result of poor access to healthcare facilities. However, the result of this study was comparable with a study conducted in Kenya which showed a mortality rate of 26.1%^10^. The median duration of ICU stay in our study was 18.46 days with a range of 2–90 days. Severe TBI and tetanus had the longest duration of stay, which was higher than studies in Jimma, with a mean length of stay of 3 days (1–7 days)^18^. And, another Nigerian study showed the mean length of an ICU stay of 3.6 days with a range of 1–16 days^19^. The reason for a longer duration of ICU stay in our study was not further analyzed but the assumption was due to the lack of rehabilitation centers in the country those patients with long-term disabilities requiring prolonged rehabilitative interventions will end up staying in ICU. However, factors resulting from prolonged critical care stay needs further research. Older patients had a significantly higher proportion of death in this study with older patients aged 60–69 (P = 0.033 70–80 (P = 0.019), which was comparable with another study conducted in Addis Ababa, which showed older patients with age > 50 had significant mortality with P < 0.01^20^.

This study also revealed places from where patients are admitted and admission diagnoses were detected as significant predictors of outcome. Showing majority of burn and non-traumatic neurosurgical patients accounted for more death compared to overall admissions respectively. This finding was almost similar to a study conducted in Nigeria and Sweden. Which showed complications of fluid and electrolyte derangement, respiratory failure, septic shock, and multiple organ failure as independent contributors to burn death. The 30 days mortality after undergoing an operation for meningioma in a Swedish study was 1.5% with (P = 0.05), higher in patients with higher grade (WHO grade II and III meningiomas) (P = 0.14)^21,22^.

The death rate of patients who underwent tumor resection and debulking was higher but in our study patient symptoms, tumor staging, and location were not considered. Which might have contributed to the unfavorable outcome for patients.

In this study, the most common ICU complication was found to be HAP (76.5%) followed by UTI (48.8%), and Bed sore (38.8%) respectively, which was higher than a study conducted in India which was 50%. But compared with other studies which documented hospital-acquired pneumonia as the most common type of HAIs in ICU settings^23–25^.

UTI was found the second most common type of ICU-acquired infection, higher than studies conducted in southern Europe, Turkey, and Iran which was reported as 35.5%. But, lower than a study conducted at Jimma University (68.71%)^24–26^. The reason for a higher number of UTIs in the ICU setup could be due to the fact that most patients were catheterized, and due to poor catheter care.

The other parameter that this study looked at was the provision of pre-hospital care on the outcome of patients. Those trauma patients who received pre-hospital care were more likely to survive than those without pre-hospital care with (AOR = 3, 7, 21.3) and (P ≤ 0.001). This finding was comparable with a study conducted in Scotland (P = 0.001)^27^.

## Conclusion and recommendations

### Conclusion

Trauma-related cases constituted the largest proportion of admissions into the AaBET hospital ICU. Road traffic accidents were the major cause of trauma-related admissions (3.5%). Trauma-related head injuries were increasing at an alarming rate in recent years, affecting predominantly showed younger ages, and the male gender. The mortality rate was found to be 21.6%, with the majority of death occurring above the age of 60.

The bed occupancy rate in this study was higher at 18.46 days.

### Recommendation

On the basis of findings obtained from this study the following recommendations were made:

### For health institutions

It is highly recommended that government institutions have a rehabilitation and convalescent center for those individuals who are left with permanent disability as a result of life-threatening injuries, as these patients had a higher median duration of ICU bed occupancy which resulted in a double burden on the setup, giving both critical and convalescent care due to lack of areas to dispose of such patients. Further research is also required in this area.

### For the road traffic safety office, and individual drivers and pedestrians

Road traffic safety and precautions need to be strictly followed as it is the major and leading cause of trauma death based on this research.

### For those health professionals who are working in ICU

Health professionals working in the institution should pay detailed attention to identifying factors associated with patient outcomes and give priority treatments for those factors that will help decrease the proportion of death.

There was poor record-keeping practice and some of the valuable information was missing from records of patients admitted to the ICU. Hence, it’s highly recommended for the institution develop a better database recording system.

### For future researchers

It will be more valuable if further studies will be conducted, on the availability of hand washing materials, and facilities including their functionality for healthcare workers, patient Families, and visitors for the practice of infection prevention.

ICU bed spacing and availability of areas to handle transmissible infectious conditions that are aerosolized and the availability of protocol and its implementation are areas that need further research in order to strengthen ICU infection prevention.

Similar prospective study on a similar setup with a larger group will help as an input to develop guidelines and protocols for ICU setups.

## Strengths and limitations

### Strengths

This study was extensive in looking at different variables in AaBET hospital ICU. It was the first of its kind in the hospital, and it could generate new ideas for further studies the study assessed the pattern of admission and the commonest diagnosis with its root cause. The study also tried to identify some of the determinants of outcome, which was important for quality office as a means of improvement in quality of the service and policymakers at large for development of guidelines and opening of rehabilitation and convalescent centers.

### Limitations

This study had a number of limitations. Being a hospital-based retrospective study, the findings are not generalizable to the general population. And, doesn’t address the cause and effect of issues raised completely. Besides, incomplete patient charts and missing of important information was the biggest challenge.

The diagnosis at ICU admission might be biased by the physician. And, vital signs were not completely registered with most charts missing the initial RBS at presentation to the ED.

The lack of similar studies in similar setups made comparison a bit difficult, and the study looked at data for two years only.

Time to compile all data and analysis also became another limitation as getting charts for review was difficult due to COVID-19 Pandemic. As most activities were diverted to the fight against the virus and were difficult to obtain charts for review in a timely manner.

## Supplementary Information

Supplementary Information.

## Data Availability

Datasets used or analyzed during the current study are available without restriction at the request of the corresponding author.

## Abbreviations

AaBET: Addis Ababa Burn, Emergency and Trauma Hospital
CT: Computerized tomography
DKA: Diabetic keto acidosis
DVT: Deep vein thrombosis
GBS: Guillain Barre Syndrome
HBM: Health belief model
HIV: Human immunodeficiency virus
ICSO: Intra cranial space-occupying lesion
IRB: Institutional Review Board
LUTH: Lagos University Teaching Hospital
MI: Myocardial infarction
RTA: Road traffic accident
SPHMMC: Saint Paul’s Hospital Millennium Medical College
SPSS: Statistical package for social science
TASH: Tikur Anbessa Specialized Hospital
WHO: World Health Organization
