# Supplementary material for: Patterns of admission and outcome of patients admitted to the intensive care unit of Addis Ababa Burn Emergency and Trauma Hospital
Source: Sci Rep. 2023 Apr 19;13:6364. doi: 10.1038/s41598-023-33437-z (PMC10113727; doi:10.1038/s41598-023-33437-z)
Supplement: Supplementary file 1 — Supplementary Information. [file 41598_2023_33437_MOESM1_ESM.docx]

Annex

MRN-------------------

Age in years ___________________

Sex

A. Male B. Female

Address

Addis Ababa

Oromia

Tigray

Amhara

c. Somali

D. SNNP

E. Afar

F. unrecorded

G. others specify

Occupation

Government employee

Private employee

Business man/ woman

Driver

Student

Farmer

Not documented

Others specify

What is the mode of transportation during ED arrival

Taxi

Ambulance

Private car

Others specify_________

Triage Category

Red

Orange

Yellow green

Vital sign during ED presentation

BP

<90/60 mmHg

90/60-139/89 mmHg

>140/90 mmHg

Un recordable

Not documented

PR

<60bpm

60-100bpm

100-140 bpm

>140 bpm

Un recordable

Not documented

SPO2 ____________

RBS in Mg/dl___________

RR____________

GCS______________

Place from where patient is admitted to the ICU

From ED

Neurosurgical ward

Orthopedics ward

General surgical ward

Recovery

Burn unit

Transferred from other centers

Others specify _____________________________

how long does the patient stayed in the ED before admission to the ICU

<1 day

1-5 days

6-10 days

11-15 days

16-20 days

21-25 days

26-30 days

>30 days

Admission category. If A go to question number 17-26,THEN 46-54 if B go to questions 18-21 then to question 29 , THEN 46-54 , if C go to question 40-45, THEN 46-54, if D go to question 33-35, THEN 46-54

Trauma

Burn

Medical

None traumatic neurosurgical conditions

Others please specify ----------------------------------

If trauma which organ system affected more than one answer is possible if A go to question 18-26, IF B,C,D,E AND F go to question 18-21 then to question 27 , if G and H go to question 18-21 then to question 37

TBI

Solid organ injury

Rib fracture

Hemothorax

Pneumothorax

Lung contusion

limb injury

pelvic fracture

spinal cord injury

others specify ______________________________

how long did it take from the time of injury to ED presentation

A.1-2 hrs.

B. 2-6 HRS

C. 6-12 HRS

D. 12-24 HRS

E. >24 HRS

did the patient get pre referral care before arrival to the ED if A go to Question 17

yes

no

what was done more than one answer is possible

TAT

IV fluid

Antibiotics

C- collar applied

Pelvic binder

Posterior gutter applied

Nothing was done

What is the cause of the injury

RTA

Falling from height

Gun shout

Assault including stone and stick injury

Others specify __________

if the patient is having traumatic Brain injury what is the severity

Severe TBI

Moderate TBI

Mild TBI

If the patient had TBI what is the CT finding

Normal

DAI

Epidural Hematoma

Subdural hematoma

Contusion

BSF

DSF

Others specify

Did the patient underwent any neurosurgical procedure after the TBI

Yes

No

If yes what was done

Hematoma evacuation

DSF elevation

Decompressive craniotomy

Others specify_______________________

What was the cause of ICU admission after patient had TBI

Monitoring

Respiratory failure

Hemorrhagic shock

Patient intubated for airway protection due to low GCS

If the cause is general surgery what type of surgical intervention was done

Emergency laparotomy

Vascular surgery

Chest tube insertion for Hemothorax

Chest tube insertion for pneumothorax

Others specify

Cause of ICU admission after the surgery

Monitoring

Respiratory failure

Hemorrhagic shock

Patient intubated for airway protection due to low GCS

If the patient sustain burn injury what type?

A. chemical

B. electrical

C. flame

D. scald

E. others specify ________________________________

Total body surface area burnt

<15%

15-30%

>30%

Reason for icu admission of the burn patient

Monitoring

Septic shock which is refractory

Respiratory failure

Others specify_____________

If the cause is neurosurgical what was the diagnosis

Astrocytoma

Ependymoma

Neuroectodermal

Arnold chiari malformation

Others specify _________

Does the patient underwent any surgical intervention if yes go to question 34 if no escape

Yes

No

If the cause if yes what was done TBI what was done

Tumor debulking and extirpation

VP shunt for obstructive hydrocephalus

EVD insertion

Biopsy taken

Others specify

What was the cause of ICU admission for the neurologic condition

Monitoring

Hemodynamic support

Patient intubated due to low GCS

Respiratory failure

Others specify ___________

If the cause is orthopedics what was the diagnosis

Pelvic fracture

Lower limb injury

Upper limb injury

Crush injury

Did the patient underwent any orthopedic intervention if yes go to 38, if not escape

Yes

No

If yes what was done

Ex fix

Amputation

Others specify

What was the reason for icu admission after orthopedic intervention

Monitoring

Hemodynamic support

Respiratory failure

Others specify

If the cause of ICU admission is medical which system is affected more than one answer is possible , if A go to q 41 , if B go to Q42,if C go to q 43,if D go to q 44, if E go to q 45

Neurologic

infectious

Respiratory

Cardiovascular

endocrine

Others specify ________________________

If the answer to question 40 is A, what is the diagnosis?

ICSOL

Peripheral neuropathy

Stroke

Others specify

if infectious what is the diagnosis

Tetanus

GBS

Botulism

Septic shock

Others specify _________________________________

If the respiratory system is affected what is the diagnosis

Type I Respiratory Failure

Type II respiratory Failure

Severe asthmatic attack

Lung cancer

Others specify _______________________________________

If cardiovascular what is the diagnosis

MI

Arrhythmia

Cardiogenic shock

Cardiogenic pulmonary edema

Others specify

If endocrine what is the diagnosis

Severe DKA

Adrenal crisis

Thyroid storm

Myxedema coma

Others specify

Comorbidities (more than one can be chosen)

Diabetes Mellitus

Hypertension

Cardiac diseases

Renal disease

Neoplasia

Was the patient intubated during the time of arrival to the ICU

Yes

No

If the answer to question number 47 is yes what was the reason for intubation

Air way protection

Respiratory failure

Hemodynamic support

Others specify -----------------

Treatment given in ICU : more than one option is possible

Mechanical vent support

Hemodynamic support

Antibiotics

Diuretics including mannitol

Seizure prophylaxis

GI prophylaxis

DVT prophylaxis

Sedation

Anti-pain

Others specify_________________

How long did the patient stayed on mechanical ventilator _________in days

Complications in the ICU

Bed sore

Icu induced delirium

Organ failure

VAP

HAP

UTI

Tracheo esophageal fistula

Others specify __________________________

Outcome of the patient

Died

Left against medical advise

Transferred to their respective wards ------------

Transferred to other center

Discharged home

If the outcome is death what was the immediate cause of death

Multi organ failure

Refractory septic shock

Brain herniation

Respiratory failure

Arrhythmia

Electrolyte imbalance

Others specify -----------------------------------

Length of stay at the ICU before death or discharge from ICU

< 1 day

1-5 days

6-10 days

11-15 days

16-20 days

21-25 days

26-30 days

>30 days

|  |  |  |
| --- | --- | --- |
